# Expression of IP-10 related to angiogenesis in uterine cervical cancers

**DOI:** 10.1038/sj.bjc.6603790

**Published:** 2007-05-15

**Authors:** E Sato, J Fujimoto, H Toyoki, H Sakaguchi, S M Alam, I Jahan, T Tamaya

**Affiliations:** 1 Department of Obstetrics and Gynecology, Gifu University School of Medicine, 1–1 Yanagido, Gifu City 501-1194, Japan

**Keywords:** IP-10, VEGF, angiogenesis, angiogenic inhibitor, uterine cervical cancer

## Abstract

Angiogenesis is essential for development, growth and advancement of solid tumours. Interferon-*γ*-inducible protein 10 (IP-10) regulates lymphocyte chemotaxis, mediates vascular pericyte proliferation and acts as an angiostatic agent, thus inhibiting tumour growth. This prompted us to study the clinical implications of IP-10 expression related to angiogenesis in uterine cervical cancers. The levels of IP-10 decreased with advancement, and the prognosis of the 30 patients with low IP-10 expression in uterine cervical cancers was poor (66%), whereas the 24-month survival rate of the other patients with high IP-10 expression was 90%. Furthermore, IP-10 levels significantly reverse-correlated with vascular endothelial growth factor (VEGF) levels in uterine cervical cancers. Interferon-*γ*-inducible protein 10 might work on suppression of angiogenesis associated with VEGF in advancement, and can be recognised as a prognostic indicator. Furthermore, IP-10 activation might be effective on the suppression of regrowth or recurrence after intensive treatment for advanced cervical cancers.

Angiogenesis is essential for development, growth and advancement of solid tumours (Folkman, 1985). Angiogenic factors from tumours induce and activate matrix metalloproteinase, plasminogen activator, collagenase and other enzymes in endothelial cells (ECs). The enzymes dissolve basement membrane of ECs, after which the ECs proliferate and migrate under the influence of angiogenic factors. Angiogenic factors induce production of integrins in the ECs. The ECs then form immature capillary tubes. Specific angiogenic factors interacting with specific angiogenic inhibitors produce specific angiogenesis in each tumour. The angiogenic factors vascular endothelial growth factor (VEGF) (Fujimoto *et al*, 1998d, 1998a, 1999a, 2001, 2004), thymidine phosphorylase identified with platelet-derived EC growth factor (Fujimoto *et al*, 1998b, 1998c, 1999b, 1999c, 2000a) interleukin (IL)-8 and basic fibroblast growth factor (bFGF) along with the angiogenic transcription factor ETS-1 (Fujimoto *et al*, 2002b) interacting with angiogenic inhibitors work on angiogenesis.

We have reported the positive correlation between microvessel counts and IL-8, and that IL-8 works as an angiogenic factor. There was a significant difference in IL-8 levels between early-stage and late-stage uterine cervical cancers. The prognosis of the higher group was radically poorer than that of the lower group. This indicates that IL-8 might be a prognostic indicator as an angiogenic factor supplied from macrophages within and around the tumour (Fujimoto *et al*, 2000b, 2002a). The expression of VEGF was correlated with microvessel density in uterine cervical cancers. The levels of VEGF and VEGF_165_ and VEGF_121_ mRNAs were remarkably higher in some stages II and III/IV adenocarcinomas of the cervix than in other cases including normal cervices. Therefore, the elevation of VEGF_165_ and VEGF_121_ might contribute to the relatively late advancing via angiogenic activity in some adenocarcinomas of the cervix (Fujimoto *et al*, 1999a, 2004). The expression of basic FGF was significantly higher in advanced primary uterine cervical cancers, regardless of histological type. This status might contribute to the acceleration of growth, invasion, and metastasis with neovascularization in advanced uterine cervical cancers (Fujimoto *et al*, 1995, 1997a, 1997b).

Specific angiogenic inhibitors have not been well known in uterine cervical cancers. Among angiogenic inhibitors, interferon-*γ*-inducible protein (IP-10) is a member of the CXC chemokine family, whose members have four highly conserved cysteine amino-acid residues, with the first two cysteines separated by one non-conserved amino-acid residue, hence the name CXC (Rollins, 1997), that has been shown to induce chemotaxis of activated T cells and to inhibit angiogenesis (Strieter *et al*, 1995). It is produced by activated monocytes, fibroblasts, ECs, epithelial cells and keratinocytes. Interferon-*γ*-inducible protein 10 binds to a seven-transmembrane G protein-coupled receptor, CXCR3, expressed on activated T cells, leading to chemotaxis (Loetscher *et al*, 1996). Downregulation of the angiostatic chemokine IP-10 has been found to be a factor in the development of idiopathic pulmonary fibrosis (Keane *et al*, 1997) and endometriosis (Yoshino *et al*, 2003). On the other hand, the overexpression of IP-10 in human lymphoma grown in nude mice leads to spontaneous regression directly related to impaired angiogenesis (Sgadari *et al*, 1996a). This has been substantiated further in model systems of human non-small cell lung cancer tumourigenesis in severe combined immunodeficiency mice (Arenberg *et al*, 2001). Retroviral gene transfer of IP-10 inhibited growth of human melanoma xenografts in an angiogenesis-dependent manner (Feldman *et al*, 2002).

This prompted us to study the expression manner of IP-10 interacting with various angiogenic factors, VEGF (Boulday *et al*, 2006), IL-8 (Elner *et al*, 2006), IL-12 (Sgadari *et al*, 1996b), TNF-*α* (Gottlieb *et al*, 2005) according to various clinical backgrounds of uterine cervical cancer patients, to know the clinical implications of IP-10 in uterine cervical cancers.

## MATERIALS AND METHODS

### Patients

Informed consent for the following studies was obtained from all patients and the Research Committee for Human Subjects, Gifu University School of Medicine. Sixty patients ranging from 26 to 78 years of age with uterine cervical cancers (22 stage-I cases, 26 stage-II cases, 12 stage-III cases; and 47 squamous cell carcinomas, 13 adenocarcinomas) underwent curative surgery at the Department of Obstetrics and Gynecology, Gifu University School of Medicine, between December 1998 and January 2004. None of the patients had received any therapy before uterine cervical cancer tissue was taken. A part of each tissue of uterine cervical cancers was snap-frozen in liquid nitrogen to determine IP-10, IL-8, and VEGF and a neighbouring part of the tissues was submitted for histopathological study including immunohistochemical staining for IP-10. The clinical stage of uterine cervical cancers was determined by International Federation of Obstetrics and Gynecology (FIGO) classification (International Federation of Obstetrics and Gynecology (FIGO) News, 1989).

### Immunohistochemistry

Sections (4 *μ*m) of formalin-fixed paraffin-embedded tissues of uterine cervical cancers were cut with a microtome and dried overnight at 37°C on a silanised-slide (Dako, Carpinteria, CA, USA). Samples were deparaffinised in xylene at room temperature (RT) for 80 min and washed with a graded ethanol/water mixture and then with distiled water. Immunohistochemical staining for factor VIII-related antigen and CD34, which are synthesised by vascular ECs, are specific for the ECs of blood vessels (Bell and Flotte, 1982) and are useful for detecting tumour angiogenesis (Weidner *et al*, 1991). The samples were soaked in a citrate buffer, and then microwaved at 100°C for 10 min. The protocol for a DakoCytomation LSAB+ System-HRP (Dako) was followed for each sample. In the described procedures, goat anti-human IP-10 (R&D Systems, Minneapolis, MN, USA), rabbit anti-factor VIII-related antigen (Zymed, San Francisco, CA, USA) and mouse CD34 (Dako) were used at dilutions of 1 : 25, 1 : 2 and 1 : 40, respectively, as the first antibodies. The addition of the first antibody, goat anti-human IP-10, rabbit anti-factor VIII-related antigen or mouse CD34, was omitted in the protocols for negative controls of IP-10, factor VIII-related antigen and CD34, respectively.

Vessels were counted in the five highest density areas at × 200 magnification (using a combination of × 20 objective and × 10 ocular, 0.785 mm^2^ field^−1^) by blinded investigators. Microvessel counts were expressed as the mean numbers of vessels in the five highest density areas (Maeda *et al*, 1996). Microvessel density was evaluated by the counting of microvessels.

### Enzyme immunoassay for determination of IP-10, IL-8 and VEGF antigens

All steps were carried out at 4°C. Tissues of uterine cervical cancers (wet weight: 10–20 mg) were homogenised in HG buffer (5 mM Tris–HCl (pH 7.4), 5 mM NaCl, 1 mM CaCl_2_, 2 mM ethyleneglycol-bis-[*β*-aminoethylether]-N,N,N′,N′-tetraacetic acid, 1 mM MgCl_2_, 2 mM dithiothreitol, 25 *μ*g ml^−1^ aprotinin, and 25 *μ*g ml^−1^ leupeptin) with a Polytron homogenizer (Kinematics, Luzern, Switzerland). This suspension was centrifuged in a microfuge at 12 000 r.p.m. (10 000 **g**) for 3 min to obtain the supernatant. The protein concentration of samples was measured by the method of Bradford (Bradford, 1976) to standardise IP-10, IL-8 and VEGF antigen levels.

Interferon-*γ*-inducible protein 10, IL-8 and VEGF antigen levels in the samples were determined by a sandwich enzyme immunoassay using a Human IP-10 Quantikine (R&D Systems), a human IL-8 Quantikine (R&D System) and a human VEGF Assay Kit-IBL (Immuno Biological Laboratories, Gunma, Japan), respectively. The levels of IP-10, IL-8 and VEGF were standardised with the corresponding cellular protein concentrations.

### Western blot analysis for IP-10

Tissues (wet weight: 10–20 mg) were homogenised in a WB–HB buffer (10 mM Tris–HCl (pH 7.4), 150 mM NaCl, 0.5% Triton X-100 and 0.2 mM phenylmethyl sulphonyl fluoride) with a Polytron homogenizer (Kinematics, Luzern, Switzerland). The protein concentrations of samples were measured by protein assay (Bio-Rad, Hercules, CA, USA). Each sample (25 *μ*l) containing 10 *μ*g of protein was added to 25 *μ*l of a sample buffer (12.5 mM Tris–HCl (pH 6.8), 2% glycerol, 0.4% SDS and 1.25% 2-mercaptoethanol) and analysed by 20% SDS–PAGE under nonreducing condition. The gel was transferred to a nitrocellulose membrane (Hybond ECL Western; Amersham, Arlington Heights, IL, USA). The membrane was blocked with 5% skim milk in a blocking buffer (20 mM Tris–HCl (pH 7.6), 137 mM NaCl and 0.1% Tween-20), incubated with rabbit anti-human IP-10 antibody (1 : 2000) (R&D Systems) for 60 min at RT, washed and then incubated with peroxidase-linked species-specific whole antibody, anti-rabbit immunoglobulin from a goat (1 : 5000; Santa Cruz Biotechnology, Santa Cruz, CA, USA) for 60 min at RT. After sequential washing with blocking buffer, PBS with 0.1% Tween 20 and PBS, signals were developed using ECL plus (GE Healthcare Bio-Sciences AB, Uppsala, Sweden) and X-ray film was exposed on the membrane at RT for 10 min. Bands on the exposed film were captured with a Fuji Image Analyzer LAS-1000 and the intensity was measured with NIH-image (National Institutes of Health, Bethesda, MA USA). The IP-10 levels as the arbitrary unit (AU) determined by Western blot analysis for IP-10 were calculated as follows: IP-10 levels by Western blotting=the intensity of IP-10 × 100/the intensity of *β*-actin.

### Statistics

Interferon-*γ*-inducible protein 10, IL-8 and VEGF were measured from three parts of the same tissue in triplicate. Statistical analysis was performed with Student's *t*-test. Differences were considered significant when *P*<0.05. Correlation evaluations between VEGF and IP-10 levels were analysed by Pearson's product–moment corelational coefficient. Positive correlation was considered significant when *P*<0.05.

## RESULTS

**Figure 1:**
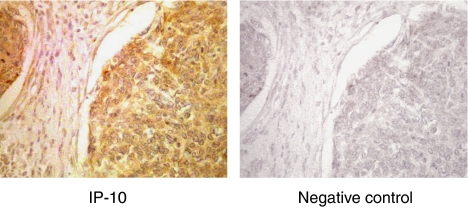
Immunohistochemical staining for IP-10 in uterine cervical cancers (original magnification × 100). A representative case of squamous cell carcinoma of the uterine cervix. Goat anti-human IP-10 (R&D Systems, Minneapolis, MN, USA) was used at a dilution of 1 : 50 as the first antibody. Dark brown staining represents positive for IP-10 antigen.

Interferon-*γ*-inducible protein 10 was diffusely located in the cancer cells, but not in the stromal cells of uterine cervical cancer tissues in all cases given, as shown in Figure 1 for a representative case (71-year-old patient, stage IIb, squamous cell carcinoma).

**Figure 2:**
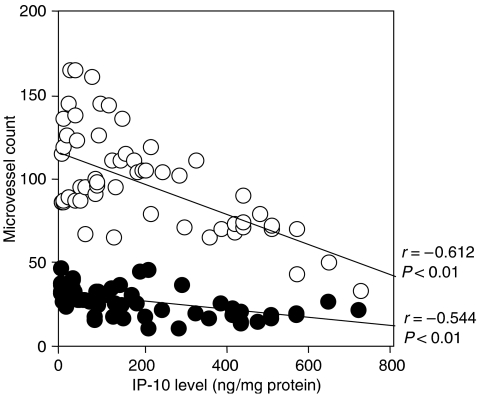
Correlation between microvessel counts and IP-10 in uterine cervical cancers. Closed circles, microvessel counts by immunohistochemical staining for factor VIII-related antigen (MVC-F8); open circles, microvessel counts by immunohistochemical staining for CD34 (MVC-CD34).

Interferon-*γ*-inducible protein 10 levels significantly reversely correlated with microvessel counts by immunohistochemical staining for factor VIII-related antigen (MVC-F8) and by staining for CD34 (MVC-CD34) (*r*=−0.544, *P*<0.001 and *r*=−0.612, *P*<0.001, respectively) as shown in Figure 2.

**Figure 3:**
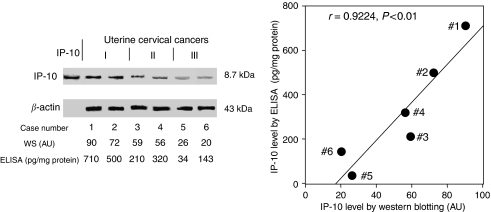
Correlation between IP-10 levels determined by Western blotting and ELISA. The bands of Western blots of the representative six cases are shown. Representative cases are shown in Figure 4 using *n*. AU, arbitrary units of IP=10.

The IP-10 levels determined by Western blotting analysis and ELISA of six representative cases are shown in the left panel of Figure 3. There was a significant positive correlation between the IP-10 levels determined by Western blotting and ELISA as shown in the right panel of Figure 3. In place of the Western blotting, ELISA can be useful to determine the IP-10 levels reliably.

**Figure 4:**
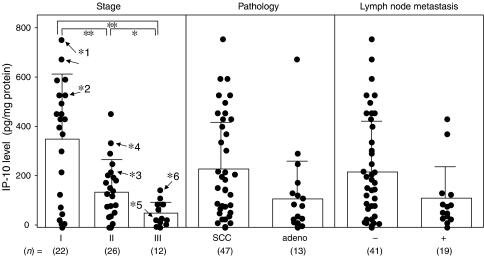
Levels of IP-10 in uterine cervical cancers classified according to clinical stage, histopathological type and lymph node metastasis. Clinical staging of uterine cervical cancer was done according to FIGO. Each level is the mean of nine determinations. ^*^P<0.05, ^**^P<0.001, *n* representative cases from Figure 3.

**Figure 5:**
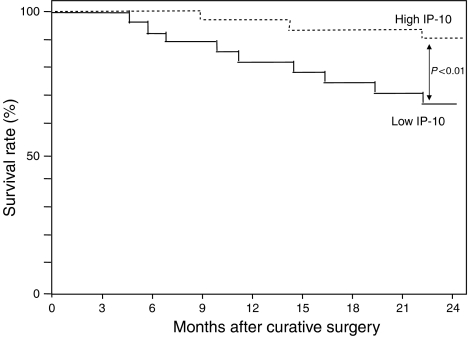
Survival rates after curative resection for uterine cervical cancers. Patient prognosis was analysed with a 24-month survival rate. High IP-10, cases with high IP-10 levels (>140 pg mg^−1^ protein), *n*=30; low IP-10, cases with low IP-10 levels (<140 pg mg^−1^ protein), *n*=30.

**Figure 6:**
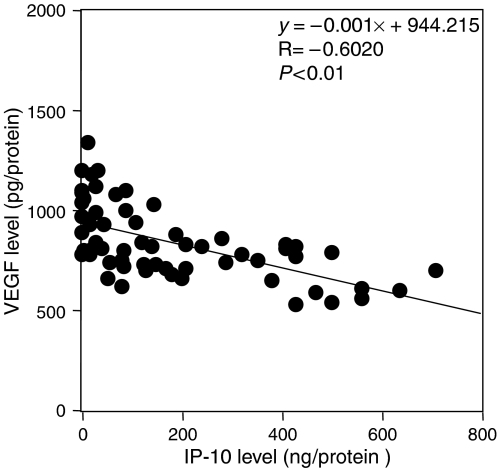
Correlation between IP-10 and VEGF in uterine cervical cancers.

Interferon-*γ*-inducible protein 10 expression did not demonstrate any significant difference according to histopathological type or lymph node metastasis, as shown in Figure 4. Interferon-*γ*-inducible protein 10 levels significantly decreased with advancement (between stages I and II, *P*<0.001; between stages I and III, *P*<0.001; and between stages II and III, *P*<0.05) as shown in Figure 4. The 60 patients were divided equally in two, at the median value of 140 pg mg^−1^ protein, to form the low IP-10 and high IP-10 groups. The prognosis of the 30 patients with high IP-10 expression in uterine cervical cancers was good, whereas the 24-month survival rate of the other 30 patients with low IP-10 expression was significantly poorer, as shown in Figure 5. Interferon-*γ*-inducible protein 10 levels reversely (*r*=−0.564, *P*<0.01) correlated with VEGF levels, as shown in Figure 6, but not with IL-8 levels in uterine cervical cancers (data not shown).

## DISCUSSION

In the present study, IP-10 reversely correlated with microvessel counts and might act as an angiostatic agent in uterine cervical cancers. Interferon-*γ*-inducible protein 10 expressed in the cancer cells decreased with tumour advancement, and downregulated IP-10 correlated with poor patient prognosis in uterine cervical cancers. This indicates that IP-10 works on angiogenesis as an angiogenic inhibitor in uterine cervical cancers. Furthermore, we were interested in how IP-10 interacts with angiogenic factors specific to uterine cervical cancers.

Interferon-*γ*-inducible protein 10 is identified as one of the TNF-regulated products in keratinocytes (Sgadari *et al*, 1996b) and it contributes to the antitumoural effects of IL-12 through the recruitment of activated T cells, NK cells, monocytes and macrophages as well as through its inhibitory effects on tumour vasculature (Elner *et al*, 2006). Human retinal pigment epithelial cells produced very high levels of IP-10 in response to either IL-1*α* or TNF-*α*, in the presence of IFN-*γ* (Boulday *et al*, 2006). Interferon-*γ*-inducible protein 10 potently inhibits angiogenesis, working in a dose-dependent manner to inhibit both IL-8 and bFGF-induced endothelial chemotaxis (Strieter *et al*, 1995). Interferon-*γ*-inducible protein 10 is expressed in some diseases in association with VEGF. It is reported that VEGF may also play a major role to augment the expression of IP-10 in T cells (Feldman *et al*, 2002). Interferon-*γ*-inducible protein 10 also inhibits the proliferation of vascular ECs via IP-10 receptor CXCR3 expressed in ECs (Lasagni *et al*, 2003). Interferon-*γ*-inducible protein 10 inhibits VEGF-induced EC tube formation concomitant with blocking motility (Bodnar *et al*, 2006). Vascular endothelial growth factor augments the effect of IFN-*γ* on the induction of IP-10 mRNA and protein expression in ECs, and Chinese hamster ovary cells designed to secrete VEGF were injected subcutaneously into the skin of nude mice and found to mediate a time-dependent increase in IP-10 mRNA (Feldman *et al*, 2002). Therefore, angiogenic factors specific to IP-10 might be IL-8, bFGF and VEGF.

Among angiogenic factors specific to IP-10, angiogenic factors specific to uterine cervical cancers seem to be IL-8 and VEGF. In the present study, IP-10 levels reversely correlated with VEGF levels, but not with IL-8, in uterine cervical cancers. The biological pathway of IP-10 induction by VEGF as a homostatic regulation might be lost with tumour advancement, resulting in drastic tumour angiogenesis that accelerates tumour advancement.
